# Not just a background: pH buffers do interact with lanthanide ions—a Europium(III) case study

**DOI:** 10.1007/s00775-022-01930-x

**Published:** 2022-02-12

**Authors:** Poulami Mandal, Jerome Kretzschmar, Björn Drobot

**Affiliations:** Institute of Resource Ecology, Helmholtz-Zentrum Dresden-Rossendorf, Bautzner Landstr. 400, 01328 Dresden, Germany

**Keywords:** Europium, Buffer, TRLFS, NMR, Stability constant

## Abstract

The interaction between Eu(III) ion and different pH buffers, popular in biology and biochemistry, viz. HEPES, PIPES, MES, MOPS, and TRIS, has been studied by solution nuclear magnetic resonance spectroscopy (NMR) and time-resolved laser-induced fluorescence spectroscopy (TRLFS) techniques. The Good’s buffers reveal non-negligible interaction with Eu(III) as determined from their complex stability constants, where the sites of interaction are the morpholine and piperazine nitrogen atoms, respectively. In contrast, TRIS buffer shows practically no affinity towards Eu(III). Therefore, when investigating lanthanides, TRIS buffer should be preferred over Good’s buffers.

**Graphical abstract:**

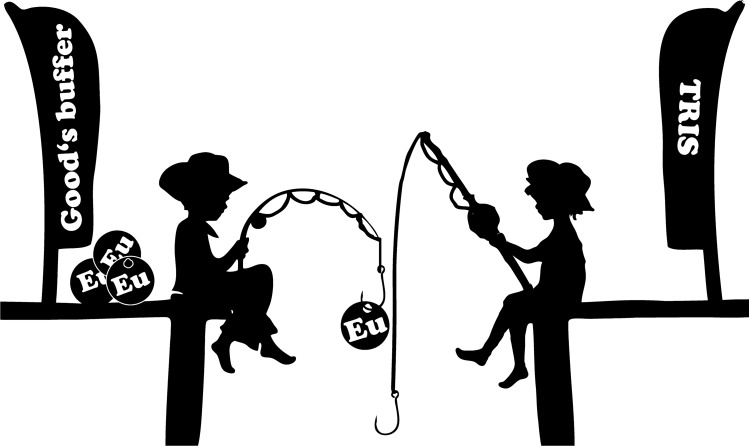

**Supplementary Information:**

The online version contains supplementary material available at 10.1007/s00775-022-01930-x.

## Introduction

A ubiquitous feature of many aqueous, chemical and biological reactions is their pH sensitivity. Chemical reactions involving complex formation [1, 2], oxidation–reduction [3, 4], catalytic reactions [5, 6] or acid–base reactions [7, 8] significantly depend on the solution’s hydrogen ion concentration. In case of, e.g., biochemical assays, a small alteration of pH can affect the reaction mechanism and consequently reaction rate, yield, or even the nature of the formed product can vary drastically [9–13]. Therefore, proper maintenance of pH is very important. To maintain a constant concentration of hydrogen ion throughout the experiments, the most effective way is to add a suitable buffer according to the required pH range.

The buffer concept is based upon the protonation–deprotonation equilibrium of a weak acid–base pair. Close to their p*K*_a_ values, buffer molecules exist simultaneously in both forms. This enables the buffer to scavenge or release protons and thereby stabilizes the pH value against changes in total proton concentration.

The use of classical buffers such as borate, citrate, succinate, phosphate, and carbonate buffers are associated with various disadvantages, e.g. incompatibility with biological conditions, precipitation upon interaction with di- or trivalent cations or direct interaction with the system under study [14, 15].

Originally intended to overcome these intrinsic drawbacks, Norman E. Good and his group developed a new group of buffers, which nowadays are known as Good’s buffers. According to Good, a buffer has to meet a number of criteria. In addition to good water solubility and non-toxicity, the buffer should not interfere with the system under study. Thus, Good et al. synthesized a class of buffers, derived from N-substituted aminosulfonic acids, which follow these criteria. The Good’s buffers are characterized by their zwitterionic structure, containing sulfonate groups as well as either morpholine or piperazine ring residues [16–18]. Good’s buffers have been extensively used for decades especially for investigations of biological systems.

However, the Good’s buffers are not devoid of drawbacks either. One well-known disadvantage is the dependence of their p*K*_a_ values on the temperature [19, 20]. This is relevant for experiments involving temperatures other than room temperature, such as thermal stability assays commonly used to assess the impact of metal binding to proteins. In addition, when these buffers are used, often no attention is paid to whether or not they affect the system under study. Neglecting such side reactions of the buffer could lead to erroneous results. Therefore, careful evaluation of the buffers’ role should be an essential part of today’s research.

In the past few years, some of the Good’s buffers have been found to interact considerably with metal ions when the buffers are used in common quantity > 10 mM [21, 22]. Few results also reported on buffer-induced effects on metal–protein systems [15]. There are a number of reports which confirm buffer complexation properties with different transition metal ions [23–30]; e.g. chromium(III)–buffer interaction has been reported to be influenced by insertion of methylene group (between the zwitterions) as well as hydroxyl group (nearby the sulfonic group) [31]. However, similar reports on lanthanide ions are sparse and contradicting [32–37].

Lanthanides came into focus of research and social awareness mainly owing to their use in modern high-tech industry. Particular interests are their magnetic properties [38, 39] as well as their outstanding luminescence [40], which makes them important for light-emitting devices [41]. Furthermore, lanthanides can be used as molecular probe; e.g*.* biological calcium binding sites can be studied by substituting calcium for europium [42]. Beside such research applications, lanthanides were considered irrelevant for biology. However, recent studies have reported native proteins optimized for lanthanide binding and specialized plants and microorganisms whose metabolic reactions rely on lanthanide ions in the active site of their enzyme [43–45]. Both in biological research and in the development of modern biotechnological lanthanide recycling processes, buffers play an important role.

**Fig. 1:**
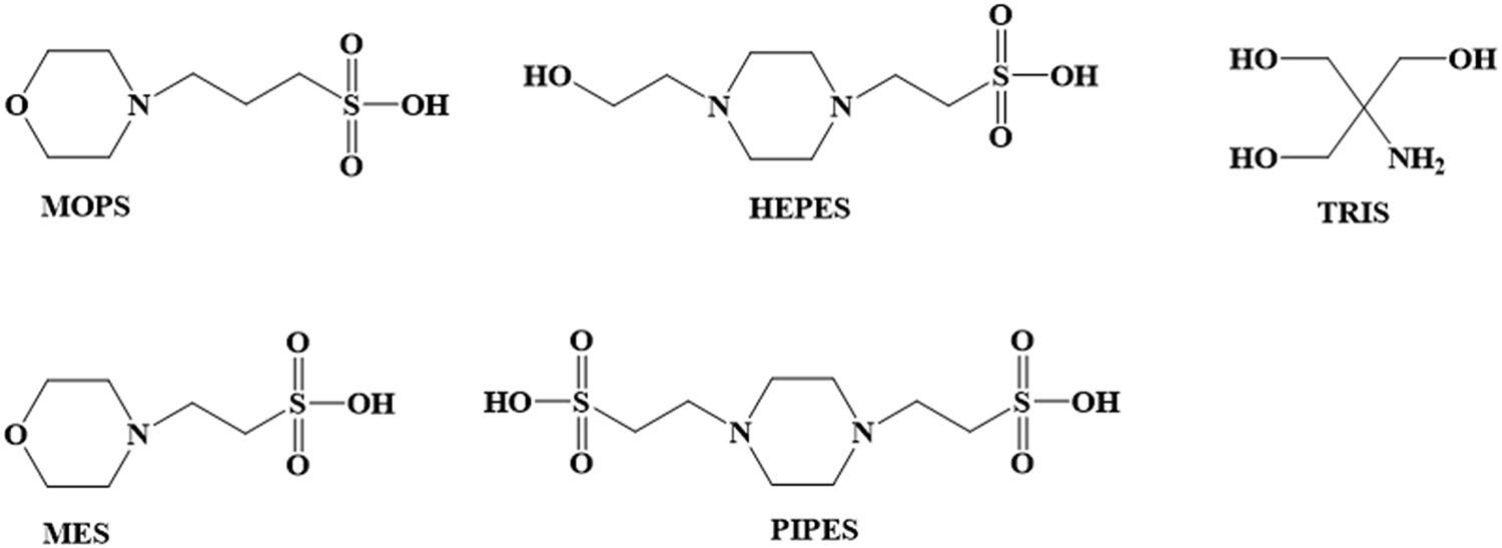
Generic structures of the buffers investigated

For our case study, we have chosen trivalent europium, Eu(III), as representative for the lanthanides. We are studying its complexation at room temperature with four frequently used Good’s buffers, i.e. MES and MOPS, containing a morpholine ring, PIPES and HEPES, containing a piperazine ring, as well as TRIS, for comparison, which contains three hydroxyl groups and its buffer relevant protonation/deprotonation occurring at a primary amine (Fig. 1). As heat capacity and reaction enthalpy of the buffers might change with changes in temperature [20], it is very important to keep the temperature fixed at room temperature throughout the experiment. With these buffers, the physiological pH range from 5.8 (MES) to 9.0 (TRIS) can be covered. However, the interpretation and decomposition of Eu(III) luminescence emission data sets is significantly complicated when Eu(III) is complexed or even present as mixed-ligand (ternary) complexes. This was the case in our previous study, investigating Eu(III) binding to lanmodulin [46] in the presence of PIPES as buffer. Consequently, we were obliged to switch to TRIS to overcome this drawback. Although the standard working range of TRIS buffer is 7.2–9.0, the pH was kept close below that range to avoid Eu(III) hydrolysis, i.e. formation of EuOH^2+^. Providing structural information and investigating the Eu(III) interaction from the buffers’ perspective, NMR spectroscopy is used to complement the time-resolved laser-induced spectroscopy (TRLFS) data treated with Parallel Factor Analyses (PARAFAC). The latter technique benefits from the outstanding luminescence properties of Eu(III) and enables emission-based metal–ligand interaction studies at low concentrations. This combination allows the determination of the complex stoichiometry and the associated stability constants, log(β). Building upon these results, we show non-negligible interaction between Eu(III) and the Good’s buffers, the latter being unmasked to be no silent spectators (when used without care).

The present study aims at sensitizing a broad scientific community to not underestimate the influence of pH buffers on metal ion interaction studies. This work has significant relevance for the broad and dynamic area of biological lanthanide studies; e.g. nowadays, there are many studies dealing with Eu(III) interaction with living cells, mainly fungi, plants, and microorganisms [47–54]. These studies are in the field of environmental science (lanthanide fate from high-tech industry), radioecology (lanthanides as non-radioactive analogs for actinides) and recycling/circular economy (classical recycling strategies are not economical). Especially the uptake of metals depends on their chemical form, as uptake mechanisms like ion channels, ABC transporter or endocytosis are speciation-dependent. Complexation with buffer may alter the bioavailability of Eu(III) and thus the interpretation of experimental results.

## Materials and methods

### Chemicals

All chemicals were used without further purification. MES, MOPS, PIPES, HEPES, TRIS, and NaCl (all with purity > 99%) were purchased from Carl Roth, and EuCl_3_∙6H_2_O (> 99.9%) from Sigma-Aldrich.

### Sample preparations

NMR samples were prepared with deuterated chemicals (all by Deutero): D_2_O, (99.98% D) as well as D_2_O solutions of both DCl (37% in D_2_O with 99% D) and NaOD (40% in D_2_O with 99% D) for adjusting pD, whereby the latter was obtained by adding 0.4 units to the pH meter reading.

Sample preparation for TRLFS was carried out at room temperature prior to the experiments. The ionic strength of NaCl was set to 150 mM for all experiments. For the luminescence measurement series, EuCl_3_ concentration was kept constant (10 µM). The buffer concentration was varied between 0 and 50 mM. The pH of the solutions was adjusted using NaOH and HCl between 6 and 7 considering two factors; first, for most of the biological reactions, pH is maintained at ~ 7 and secondly, the pH range was chosen in such a way so that Eu(III) hydrolysis is avoided. For MES (p*K*_a_ = 6.27) and PIPES (p*K*_a_ = 7.14) pH was adjusted to 6.5; whereas for HEPES (p*K*_a_ = 7.56) and MOPS (p*K*_a_ = 7.18) pH was adjusted to 7.0 as for TRIS (p*K*_a_ = 8.07); p*K*_a_ values taken from [55].

### Nuclear magnetic resonance (NMR)

NMR studies comprise one- and two-dimensional experiments, the latter of which being (via-bond) ^1^H,^1^H correlation spectroscopy (COSY) and (through-space) ^1^H,^1^H nuclear Overhauser effect spectroscopy (NOESY). Data were obtained at (25 ± 1) °C on Agilent DD2-600 14.1 T and DD2 MR-400 9.4 T systems, operating at ^1^H resonance frequencies of 600 and 400 MHz, respectively, using 5 mm oneNMR^™^ probes. The solvent (HDO) signal was suppressed by a pre-saturation sequence, with a 2 s selective pulse on the HDO resonance, the latter depending on [Eu^3+^] and pD. Spectra were referenced externally relative to the Si(Me)_3_ signal of TMSP with *δ* = 0 ppm. The double-quantum filtered (DQF) COSY was recorded using 1024 × 128 data points in *F*_2_ and *F*_1_ and 8 acquisitions in *F*_1_, zero-filled to 2048 × 512 real data points, applying cosine bell and cosine-squared bell window functions to *F*_2_ and *F*_1_, respectively. The NOESY was acquired with 1024 × 256 data points in *F*_2_ and *F*_1_ and 16 transitions per *F*_1_ increment, using 1 s pre-saturation and a mixing time of 500 ms. In both dimensions the FIDs were multiplied by a cosine bell and zero-filled to obtain 2048 × 1024 real data points in *F*_2_ and *F*_1_, respectively.

For NMR experiments, initially NMR data were collected for the free buffers individually and then the buffer was titrated with increasing concentration of EuCl_3_ in D_2_O. NMR data were acquired at two different pH values (3 and 4.5) to check the effect of protonation-deprotonation at the N or O center of the buffer upon complexation.

### Time-resolved laser-induced fluorescence spectroscopy (TRLFS)

All buffer titration series were performed in triplicate using TRLFS. Laser pulses (5 ns, 2 mJ/pulse) with an excitation wavelength of 394 nm were generated using a pulsed Nd: YAG-OPO laser system (Powerlite Precision II 9020, PANTHER EX OPO, Continuum). Eu(III) luminescence was collected by a glass fiber at 90° configuration and detected by a Andor iStar ICCD-camera (Lot-Oriel Group) connected with the rear end of the spectrograph (Oriel MS 257, 300 lines per mm grid). The gate width of the camera was set to 300 μs. The luminescence decay was detected by measuring 25 different temporal offsets to the laser beam using a linearly increasing step size (3 + 3*x* [µs], 1.5 < *x* < 325.5) (7.5–979.5 µs).

### Parallel factor analysis (PARAFAC) applied to TRLFS spectra

Luminescence spectroscopic data sets were analyzed using parallel factor analysis (PARAFAC, implemented as N-way toolbox for MATLAB [56] with following modifications as described elsewhere [42]. The luminescence decays of individual species were constrained to be exponential and the species distribution had to reflect a speciation of the buffer–metal system. The p*K*_a_ values of the buffers [55] were considered for the speciation calculation to extract complex stability constants log(β).

## Results and discussion

Both NMR and TRLFS provide their intrinsic strengths and drawbacks, which make them an ideal complementary pair of techniques. In principle, NMR spectroscopy itself is able to determine structural and thermodynamic parameters of a system. Unfortunately, relative high analyte concentrations are needed and the paramagnetism of most lanthanide ions complicates the interpretation of the various effects. In contrast, TRLFS does not provide direct structural information. The strength of fluorescence lies in its extremely high sensitivity, enabling studies in the µM concentration regime. Here, we started our investigations using NMR for structure elucidation, followed by TRLFS studies for the determination of the complex stability constants.

### NMR

To address the binding sites, and therefore to comprehend Eu(III)-induced effects on the spectra, correct assignment of the buffers’ signals is crucial. Normally the buffer is a background component used in high concentrations. Its signals are considered uninteresting and, at best, do not overlap with the actual signals of interest. It is thus not surprising that there is virtually no literature on buffer signals.

One of the scarce sources for reference data is the Biological Magnetic Resonance Data Bank (BMRB) [57]. However, details accompanying the spectra are somewhat confusing. Stated with all buffer spectra is a solution pH of 7.4 while the samples are prepared in (pure) D_2_O solution, hence requiring for a pD value or some kind of correction. Moreover, the solution pH value is stated throughout as 7.4; although the buffers have different p*K*_a_ values—which is what they are designed for. Accordingly, the respective pH (pD) windows where the buffers are supposed to act as such may be different from the given pH of 7.4, and hence the spectra may be different for the corresponding pH window of usage.

An intrinsic drawback of studying the buffers’ NMR spectra is their sensitivity to intra- and intermolecular dynamics as accompanied not only with the supposed acid–base reaction, but also proton–proton, proton–deuteron, and deuteron–deuteron exchange reactions at functional groups (far below the corresponding p*K*_a_ value) as well as conformational changes of the respective morpholine or piperazine rings. Things get even worse when Eu(III) comes into play, as the Eu(III)’s paramagnetism causes NMR signal broadening owing to effective relaxation and, additionally, site-exchange reactions between free and Eu(III)-coordinated buffer molecules.

Consequently, experiments for principal NMR signal assignment were carried out at low pD and, building upon that assignment, the pD-dependent spectral behavior was studied to comprehend the various intra- and intermolecular processes.

**Fig. 2:**
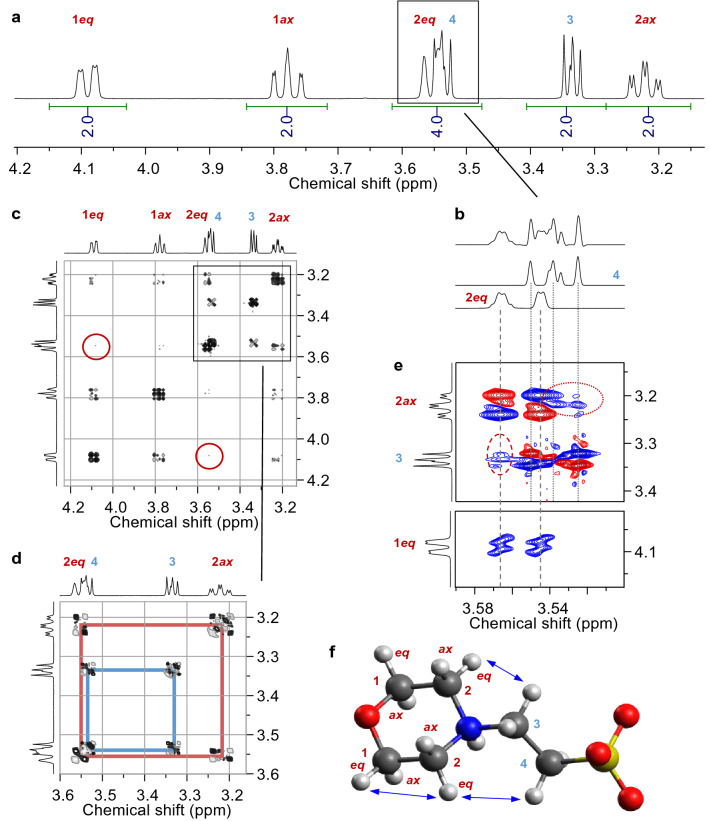
**a**–**e** 600 MHz NMR spectra of 100 mM MES buffer in pD 1 D_2_O solution at 25 °C. ^1^H NMR spectrum without window function multiplication, showing signal integrals (**a**), and indicated region processed with exponential line broadening of −1.5 Hz and Gaussian line broadening of +0.8 Hz for resolution enhancement as well as sub-spectra obtained from deconvolution (**b**). Double-quantum filtered COSY spectrum (**c**) and corresponding magnification (**d**). H, H NOESY spectrum acquired with 500 ms mixing time (**e**). Signal assignment is according to the labeling given with the molecular structure in f (C, dark gray; H, light gray; N, blue; O, red; S, yellow), where ax and eq, respectively, denote axial and equatorial positions, and arrows indicate non-trivial NOE contacts depicted in (**e**)

**Fig. 3:**
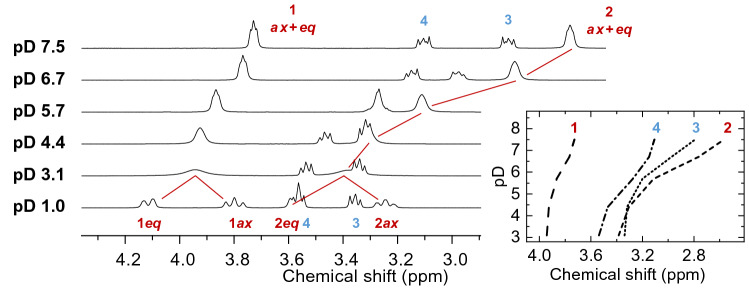
400 MHz ^1^H NMR spectra of 100 mM MES buffer in D_2_O solution at 25 °C obtained at different pD values as indicated. The inset shows the graphical evaluation of pD-dependent chemical shifts

NMR spectral (pH-dependent) characteristics of the six-membered N-heterocyclic ring containing Good’s buffers are exemplarily discussed for MES in Figs. 2 and 3. The following issues had to be clarified: discrimination between axial and equatorial positions of protons in the heterocyclic ring fragment (denoted by *ax* and *eq,* respectively) as well as discrimination between the chain fragment’s methylene groups adjacent to the nitrogen and adjacent to the sulfonate group (in case of MES labeled 3 and 4, respectively).

Figure 2 shows NMR spectra of a MES solution at pD 1, resulting in sharp, well-resolved signals consistent with lacking dynamics. Only two of the six signals overlap as implied by the signals’ integrals, whereby each signal has an integral of two, corresponding to four different ring hydrogen atoms being pairwise equivalent by symmetry (two AA’XX’ spin systems). Since in the ring fragment these methylene groups are fixed, the two hydrogen atoms on one carbon atom are distinct and hence show *geminal* coupling (via two bonds, ^2^*J*). That is, each of the ring’s equatorial and axial hydrogens on one side of the plane going through O, N, C3, C4, and S are mirrored. Although being able to rotate freely, the two hydrogens of either methylene groups in the chain fragment are enantiotopic, hence both methylene groups constitute an AA’BB’ spin system.

According to the Karplus relationship [58], the *vicinal* coupling constant (via three bonds, ^3^*J*) depends on the dihedral angle between the coupling protons, whereby ^3^*J* is largest for dihedral angles close to 0° (~ 11 Hz) and 180° (~ 13 Hz), and is smallest (~ 2 Hz) for a dihedral angle of 90°. Therefore, axial–axial coupling (^3^*J*_1*ax*,2*ax*_) is large by about 13.0 Hz, which is virtually the same magnitude as |^2^*J*| with 13.1 Hz, while axial–equatorial couplings are much smaller (^3^*J*_1*ax*,2*eq*_ = 2.3 Hz, ^3^*J*_2*ax*,1*eq*_ = 3.7 Hz). Consequently, the signals associated with the protons in axial position are doublets of triplets as they exhibit two large couplings (^2^*J* and ^3^*J*_*ax*,*ax*_) and each one small coupling (^3^*J*_*ax*,*eq*_), whereas the signals due to protons in equatorial position are doublets of doublets (*dd*), each showing a larger (^2^*J*) and a smaller (^3^*J*_*ax*,*eq*_) splitting. After application of appropriate window functions, the *dd* character of signal 2*eq* is better seen (Fig. 2b). The equatorial-equatorial coupling via bonds is so weak that it can barely be detected in the COSY spectrum (red circles in Fig. 2c) and not at all in the one-dimensional ^1^H spectrum since the coupling constant is smaller than the line width. However, the dipolar ‘through-space’ coupling between the equatorial hydrogen atoms 1*eq* and 2*eq* are nicely observed in the NOESY spectrum (*cf.* Figure 2e and arrows in Fig. 2f).

Assignment of the methylene groups in position 3 and 4 to the signals observed at *δ* 3.54 and 3.33 ppm simply by chemical shift is not straightforward since both CH_2_ groups are adjacent to an electron withdrawing nitrogen atom or sulfonate group, respectively. The other non-trivial NOE contacts detected between hydrogens 2*eq* and both methylene groups 3 and 4 (ellipses in Fig. 2e) do not allow for unambiguous assignment either.

However, a coarse proof-of-principle titration series (Fig. 3) reveals the correct assignment. Since sulfonic acid groups are very strong acids, for the considered spectra (pD 3.1–7.5) the former is deprotonated and the site of interest is the morpholine nitrogen undergoing deprotonation. Therefore, nuclei in direct vicinity are sensitive probes for that particular structural change. That is, the signals associated with CH_2_ groups 2 and 3 show much stronger pD-dependent chemical shift changes than do the remote sites 1 and 4. Thus, the protons on C3 are somewhat more shielded than those bound to C4, testifying the signal assignment given with the NMR spectra.

The titration series exhibits significant changes in the spectral appearance, i.e., six signals observed at pD 1 but as of pD 3.1 (for 400 MHz) the number of signals reduces to four. The two bottom spectra in Fig. 3 nicely demonstrate that the signals associated with the ring fragment’s methylene groups coalesce, averaging the signals of the axial and equatorial positions, resulting in one apparent signal each for sites 1 and 2, respectively. This observation is caused by fast interconversion between different conformations of the morpholine ring (ring flip). Considering the resonance frequency differences (Δ*ν*) of the axial and equatorial signals for an interconversion slow on the corresponding 400 MHz NMR time scale, viz. Δ*ν* (1*ax*, 1*eq*) ~ Δ*ν* (2*ax*, 2*eq*) ~ 130 Hz at pD 1, the interconversion rate *k* can be calculated for the coalescence spectrum (pD 3.1) by means of *k* = 2.22 Δ*ν*, yielding a rate of about 300 ring flips per second. Upon further increasing pD, *k* approaches the fast NMR exchange regime so that the merged signals become narrower, and now representing time-averaged signals of all coexisting (acid and base) species and their individual conformers.

A minor contribution to line broadening and presumably correlated with the pD-dependent ring inversion rate is the exchange of the kinetically labile N–H hydrogen (N–D deuterium) with the solvent’s protons (deuterons) even far below the thermodynamic deprotonation constant, p*K*_a_, being 6.27 for the MES buffer [55]. To avoid the difficulties arising from intramolecular dynamics and exchange reactions, ^1^H NMR spectra of Eu(III)-containing solutions were measured far below the buffers’ corresponding p*K*_a_, i.e. at pH 3 and 4.5. These conditions allow for better discrimination between pH-associated and Eu(III)-induced effects.

**Fig. 4:**
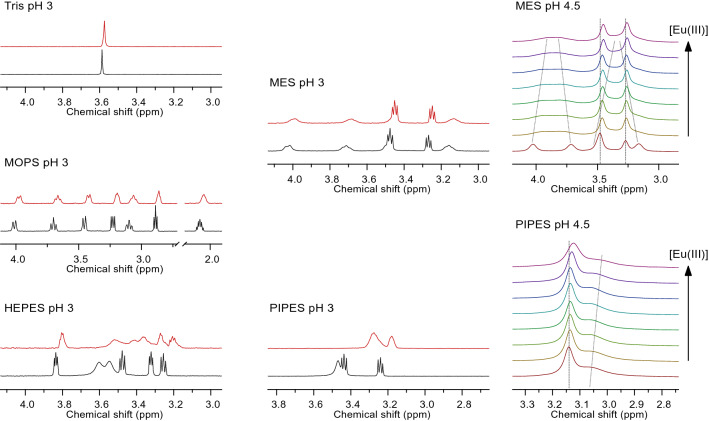
600 MHz ^1^H NMR spectra obtained from 1 mM buffer solution for pH values as stated with the spectra, where the bottom spectra (black) show the buffer only, and the top spectra (red) show the buffer in presence of 40-fold excess Eu(III), respectively. Depicted on the right are exemplary titration series at pH 4.5, with [Eu(III)] from bottom to top (in mM): 0; 0.1; 0.2; 0.5; 1.0; 2.0; 5.0; 10.0

The spectra given in Fig. 4 reveal three major observations. First, lanthanide-induced effects (LIEs), i.e. signal shifts and broadenings, occur owing to the interaction between buffer and the paramagnetic Eu(III)’s f-electrons’ unpaired spins, with the interaction being dipolar (pseudo-contact contribution). Second, for the Good’s buffers, the signals associated with the protons of the six-membered ring show strongest LIEs while those associated with the aliphatic chains show much less LIEs. Apparently, the site of interaction is located with the donor atoms in the morpholine and piperazine rings, respectively (*cf.* pH 4.5 titration series, Fig. 4, right); if the coordination of the Europium(III) was with sulfonic acid group of the buffer, the chain protons would have experienced more LIE rather than the ring protons; which also supports Eu(III) coordination at the N-atom of the ring. Third, especially upon comparison of the magnitude of LIEs at pH 3 for the different buffers, an interesting observation becomes apparent. The buffers show interaction with Eu(III) increasing in the following order: TRIS < HEPES ≈ MES ≈ MOPS << PIPES. This is due to the buffers’ speciation at pH 3, viz. TRIS is cationic (RNH_3_^+^), while HEPES, MES, and MOPS are net-neutral (R_3_NH^+^/SO_3_^−^), and PIPES is anionic (R_3_NH^+^/2×SO_3_^−^). Apparently, electrostatic forces play an important role.

### TRLFS data

**Fig. 5:**
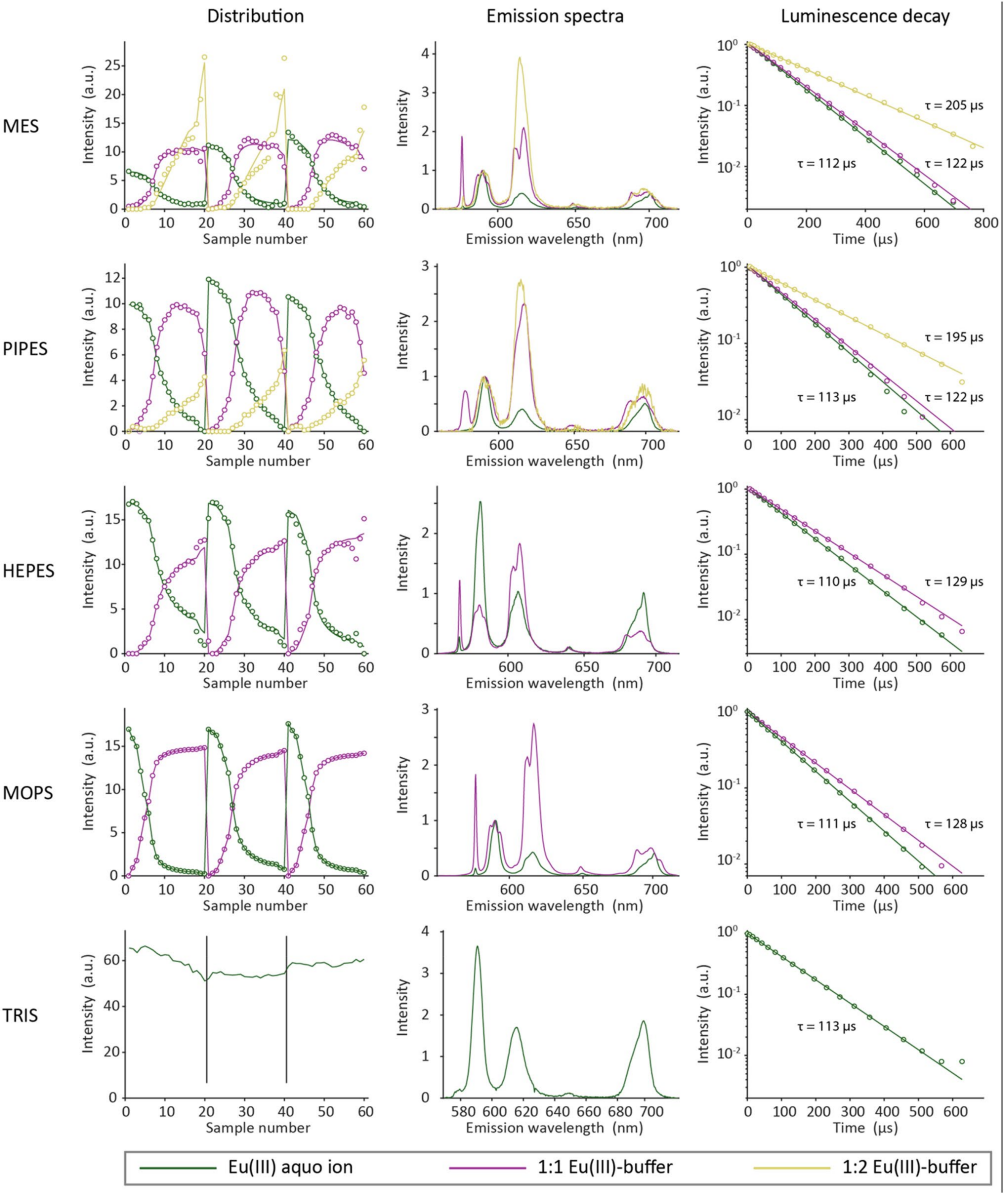
Species distribution (triplicate of 20 sample series, left column), emission spectra (middle column), and luminescence decay curve (right column) for the Eu(III)–buffer titration. The triplicate series (20 samples each, 60 in total) of all buffers were analyzed simultaneously. Therefore, the distribution in the left column was plotted against the sample number and not against the concentration

As previously described, TRLFS data were analyzed using PARAFAC. Therefore, entire datasets of the titrations were analyzed simultaneously. As a result, one gets a unique set of emission spectrum, lifetime, and distribution for every chemical species within the data series. Deconvolution results are shown in Fig. 5. In good agreement with NMR results, we see that, except for TRIS, for which no complexation is observed even for maximum buffer concentration of 10 mM at pH 6.8, all the Good’s buffers form complexes with Eu(III). Interestingly, complexes of higher stoichiometry (1:2 complex, yellow) were found only for MES and PIPES, whereas MOPS and PIPES form only 1:1 Eu(III)–buffer complexes (magenta).

As the emission spectra depict, the green spectra are associated with the symmetric Eu(III)–aquo complex, where the ^5^D_0_ → ^7^F_0_ transition is symmetry-forbidden according to Laporte selection rule and the spectra are dominated by the ^5^D_0_ → ^7^F_1_ transition. In the TRIS system, this is the exclusive species. The weak ^5^D_0_ → ^7^F_0_ transition observed in some of the aquo ion spectra can be explained by some contribution of dissolved carbonates or onset of Eu(III) hydrolysis at pH 7. Observation of this transition indicates reduced coordination symmetry around the Eu(III).

Upon complexation, Eu(III) luminescence is characterized by a change of the F_1_ and F_2_ transition’s intensity ratio (F_1_/F_2_). Eu(III) complexed by just water ions (aquo ion) is the only Eu(III) environment known to provide F_1_/F_2_ > 1. Therefore, the F_2_ transition is called hypersensitive and is commonly used as marker for Eu(III) complexation. In all our studied cases except for TRIS, F_1_/F_2_ was significantly altered upon Eu(III)–buffer complexation. Moreover, due to the removal of H_2_O molecules from Eu(III) hydration shell during complex formation, symmetry at the Eu^3+^ center is lowered and electronic transition to ^7^F_0_ becomes allowed and gives a sharp peak at wavelengths around 580 nm (see magenta spectra in Fig. 5). Interestingly, for all of the Good’s buffers studied, the ^5^D_0_ → ^7^F_0_ transitions becomes as intense as the ^5^D_0_ → ^7^F_1_ transition. Upon continuation of MES and PIPES addition, further H_2_O molecules are replaced at the Eu^3+^ center yielding a 1:2 Eu(III)–buffer complex. For electrostatic reasons, the second buffer molecule complexes on the opposite side of the Eu(III) center. This partly restores symmetry, resulting in much weaker ^7^F_0_ transitions for the 1:2 complexes (yellow spectra, Fig. 5).

From the luminescence decay curve the lifetime of Eu^3+^-aquo complex is calculated as *τ*_aquo_ ~ 110 µs. For the 1:1 complexes, the average lifetime is *τ*_1:1_ ~ 125 µs, and for the 1:2 complexes of Eu(III) and MES or PIPES, *τ*_1:2_ is 203 ± 4 µs and 195 ± 5 µs, respectively. This correlates with water molecule stripping from the Eu(III) ion upon buffer complexation. The Eu(III) luminescence lifetimes ($\tau$ in ms) can be used to calculate the number of coordinating water molecules in the first hydration sphere using the Horrocks equation [59] modified by Kimura and Choppin [60].

$${n}_{{\mathrm{H}}_{2}\mathrm{O}} \pm 0.5=\frac{1.07}{\tau }-0.62.$$

The lifetime of ~ 125 µs correlates to 8 remaining water molecules, while the longer lifetimes indicate complexes with additional 3–4 water molecules stripped.

**Fig. 6:**
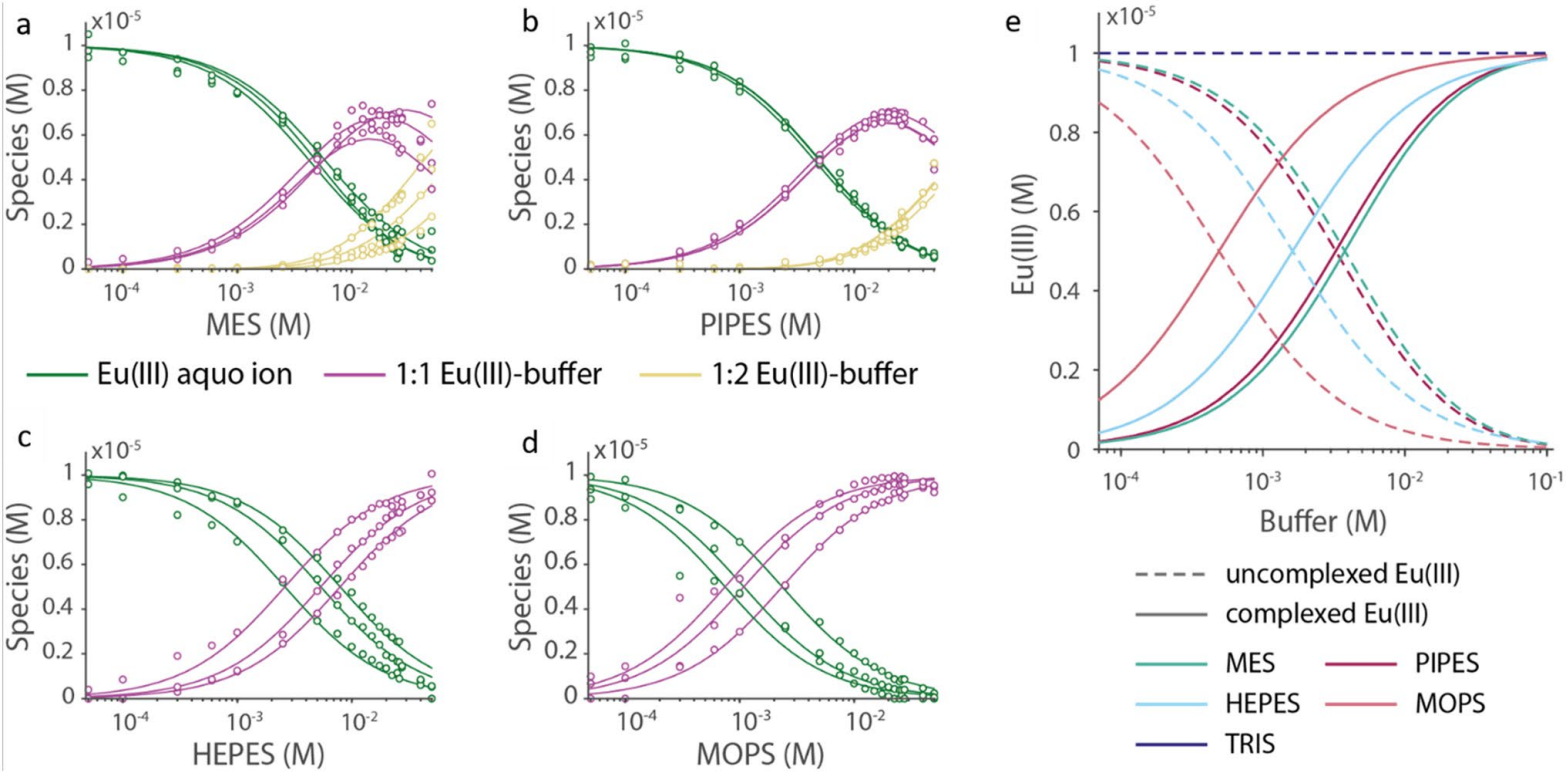
**a**–**d** Species distribution of Eu(III)–buffer complex as derived from triplicate TRLFS experiments (symbols: experiment; lines: speciation fit); **e** speciation of the Eu(III)–buffer system using log(β) values for different buffers calculated at pH 7. In all cases, total [Eu(III)] = 10 µM

Based on the PARAFAC species distribution, a speciation of the Eu(III)–buffer system can be calculated [42]. This is shown for all complexing buffers in Fig. 6. The concentration of the uncomplexed Eu(III) aquo ion decreases to less than 80% (< 2 µM) when buffer concentration exceeds 10 mM for total Eu(III) concentration of 10 µM.

The stability constants (Table 1) of the formed complexes are based on the structural information gained from NMR. With this structural information we were able not only to determine the Eu(III) buffer affinity for defined experimental conditions (e.g. buffer concentration and pH), but also the complex stability constant log(β). This enables a calculation of the speciation even for deviating conditions, allowing for comparability of the speciation of all buffers at pH 7 (Fig. 6e). For a better comparison, the 1:1 and 1:2 complexes of MES and PIPES were summed up. The fraction of complexed Eu(III) at pH 7 decreases in the order MOPS > HEPES > PIPES > MES. Presence of one extra CH_2_ group in case of MOPS buffer could be the reason for its higher affinity towards Eu(III) ion than MES buffer [31]. The Eu(III) complexes of HEPES and PIPES reveal almost identical stability constants. Even though the complex formation constant is not very high, low-affinity interactions become important at higher concentration ranges as is common for buffers. In a typical buffer concentration regime (10 mM) more than 80% of the 10 µM total Eu(III) is complexed by the buffer as demonstrated in Fig. 6e. In systems with mid-affinity ligands, this effect must not be ignored.

**Table 1:** Log(β)_1:1_ and log(β)_1:2_ values for the Eu(III)–buffer complexes

Reaction	log(β)_1:1_	log(β)_1:2_
Eu^3+^ + HHEPES → [Eu-HEPES]^2+^ + H^+^	− (4.10 ± 0.24)	–
Eu^3+^ + HPIPES → [Eu-PIPES]^2+^ + H^+^ Eu^3+^ + 2 HPIPES → [Eu-PIPES_2_]^+^ + 2H^+^	− (4.1 ± 0.1)	− (9.4 ± 0.1)
Eu^3+^ + HMES → [Eu-MES]^2+^ + H^+^ Eu^3+^ + 2 HMES → [Eu-MES_2_]^+^ + 2H^+^	− (3.7 ± 0.1)	− (8.5 ± 0.2)
Eu^3+^ + HMOPS → [Eu-MOPS]^2+^ + H^+^	− (3.5 ± 0.3)	–

There are several reports where stability constants for different La(III) complexes with various bioligands have been reported, but to the best of our knowledge, there is no such report where stability constants for the Eu–buffer complexes have been presented.

## Conclusion

Eu(III)–buffer interaction was analyzed using NMR and TRLFS techniques and stability constants for the interaction were calculated. The provided complex stability constants are based on complex structure and buffer p*K*_a_, so these thermodynamic parameters can be used to calculate speciation of Eu(III)–buffer-systems with different composition (metal and buffer concentrations and pH). The results indicate that all the chosen buffers except TRIS interact significantly with Eu(III). Depending on the log(β) values for the 1:1 complex, the extent of complexing capacity can be considered as MOPS > MES > HEPES = PIPES. The overall interaction of the buffers with Eu(III) is comparatively weak, but usually buffers are used in quite high concentrations (> 10 mM), which makes them effective lanthanide scavengers. Obviously, metal ion complexation by the buffer is not negligible especially when the molecule of interest (biomolecule, protein, receptor etc.) shows only weak interaction with the metal. Here, a detailed knowledge of its complexation properties is mandatory before using these buffers to maintain pH. From the lanthanide point of view, TRIS can be used without any ambiguity, which is definitely advantageous, bearing in mind TRIS’ p*K*_a_ dependence on temperature. In interaction studies of strong lanthanide binders, the comparatively weak buffer–lanthanide interaction may not interfere very much. However, if luminescence emission is the experimental readout, one has to be aware that the Eu(III) aquo ion is not the background species. If, e.g., isothermal titration calorimetry is performed, parameters such as reaction enthalpy will be incorrectly determined if the background reaction between buffer and metal ion are not taken into account.

In general, the buffers’ affinity toward Eu(III) increases with pH and becomes significant at the latest when pH approaches the buffer’s p*K*_a_ value. Since electrostatic repulsion additionally impedes buffer–Eu(III) interaction, the recommended pH range is below the buffer p*K*_a_, as it combines (still) good buffering capacity and reduces unintended buffer–Eu(III) complexation.

Moreover, Eu(III) luminescence spectra are representative for their corresponding binding motif. Thus, Eu(III) emission spectra can principally provide structural information. This information is usually not readily available and therefore has often been determined by spectra comparisons. In our report, we provide characteristic emission spectra with additional extensive structural analysis. We strongly believe that our data can thus act as reference spectra for similar Eu(III) complex environment.

## Supplementary Information

Below is the link to the electronic supplementary material.

Supplementary file1 (PDF 199 KB)

## Abbreviations

**HEPES**: 2-[4-(2-Hydroxyethyl)piperazine-1-yl]ethanesulfonic acid
**MES**: 2-(N-morpholino)ethanesulfonic acid
**MOPS**: 3-Morpholinopropane-1-sulfonic acid
**PIPES**: 1,4-Piperazinediethanesulfonic acid
**TRIS**: Tris(hydroxymethyl)aminomethane
**NMR**: Nuclear magnetic resonance (spectroscopy)
**TRLFS**: Time-resolved laser-induced fluorescence spectroscopy
