# Supplementary material for: Not just a background: pH buffers do interact with lanthanide ions—a Europium(III) case study
Source: J Biol Inorg Chem. 2022 Feb 12;27(2):249–60. doi: 10.1007/s00775-022-01930-x (PMC8907096; doi:10.1007/s00775-022-01930-x)
Supplement: Supplementary file 1 — Supplementary file1 (PDF 199 KB) [file 775_2022_1930_MOESM1_ESM.pdf]

Supporting Informations

Not just a background: pH buffers do interact with lanthanide ions – a Europium(III) case study

Poulami Mandal[1], Jérôme Kretzschmar[1] and Björn Drobot[1*]

[1]Helmholtz-Zentrum Dresden-Rossendorf, Institute of Resource Ecology,
Bautzner Landstr. 400, 01328 Dresden, Germany.

* Corresponding author: Dr. Björn Drobot, b.drobot@hzdr.de

Table S1. Pipetting scheme

Sample	V_{EuCl_3} (µL)	$V_{Buffer\ total}$ (µL)	$V_{Buffer\ added}$ (µL)	C_{Buffer} (µM)
1	2000	0.00	0.0	0
2	2000	2.00	2.0	50
3	2000	4.01	2.0	100
4	2000	12.07	8.1	300
5	2000	24.29	12.2	600
6	2000	40.82	16.5	100
7	2000	105.26	64.4	2500
8	2000	222.22	117.0	5000
9	2000	352.94	130.7	7500
10	2000	500.00	147.1	10000
11	2000	666.67	166.7	12500
12	2000	857.14	190.5	15000
13	2000	1076.92	219.8	17500
14	2000	1333.33	256.4	20000
15	2000	1636.36	303.0	22500
16	2000	2000.00	363.6	25000
17	1636.4	2000.00	0.0	27500
18	1333.3	2000.00	0.0	30000
19	500.0	2000.00	0.0	40000
20	0.0	2000.00	0.0	50000

Table S2. Instrument setup

Linear increasing step size	3+3*x	µs
Initial delay	0.5 (+7)	µs
Gate width	300	µs
Slit width	100	µm
Accumulations	250	
Kinetic series length	25	
Gain	3000	
